# Everyday functioning in young onset dementia: differences between diagnostic groups

**DOI:** 10.1002/alz.70711

**Published:** 2025-09-24

**Authors:** Emma Weltings, Merel C. Postema, Maureen van Dam, Mark A. Dubbelman, Mukrabe E. Tewolde, Flora H. Duits, Afina W. Lemstra, The LEADS consortium, Bradford C. Dickerson, Maria C. Carrillo, Gil D. Rabinovici, Dustin B. Hammers, Wiesje M. Van der Flier, Liana G. Apostolova, Yolande A. L. Pijnenburg, Sietske A. M. Sikkes

**Affiliations:** ^1^ Alzheimer Center Amsterdam Department of Neurology Vrije Universiteit Amsterdam, Amsterdam UMC Amsterdam The Netherlands; ^2^ Amsterdam Neuroscience, Neurodegeneration Amsterdam The Netherlands; ^3^ Center for Alzheimer Research and Therapy Department of Neurology Brigham and Women's Hospital, Harvard Medical School Boston Massachusetts USA; ^4^ Department of Neurology Massachusetts General Hospital, Harvard Medical School Boston Massachusetts USA; ^5^ Medical & Scientific Relations Division Alzheimer's Association Chicago Illinois USA; ^6^ Department of Neurology University of California – San Francisco San Francisco California USA; ^7^ Department of Neurology Indiana University School of Medicine Indianapolis Indiana USA; ^8^ Department of Epidemiology and Data Science Vrije Universiteit Amsterdam, Amsterdam UMC Amsterdam The Netherlands; ^9^ Department of Radiology and Imaging Sciences Center for Neuroimaging Indiana University School of Medicine Indianapolis Indiana USA; ^10^ Department of Medical and Molecular Genetics Indiana University School of Medicine Indianapolis Indiana USA; ^11^ Faculty of Behavioral and Movement Sciences Department of Clinical Neuro and Developmental Psychology, Vrije Universiteit Amsterdam Amsterdam The Netherlands

**Keywords:** alzheimer's disease, amsterdam IADL questionnaire, behavioral variant frontotemporal dementia, dementia with lewy bodies, posterior cortical atrophy, primary progressive aphasia, young onset dementia

## Abstract

**BACKGROUND:**

The aim of this study was to examine differences in Instrumental Activities of Daily Living (IADL) among young‐onset dementia (YOD) diagnoses.

**METHODS:**

Participants were included from Amsterdam Dementia and Longitudinal Early‐Onset Alzheimer's Disease (LEADS) cohorts, with diagnoses of typical Alzheimer's disease (AD), behavioral variant frontotemporal dementia (bvFTD), primary progressive aphasia (PPA), posterior cortical atrophy (PCA), or dementia with Lewy bodies (DLB) established in multidisciplinary meetings. We compared overall IADL scores and item level scores between groups using multiple regression analyses, adjusted for cohort, demographics, and disease severity.

**RESULTS:**

We included 582 YOD patients (58.4 ± 4.2 years; 59%F), with overall moderate IADL problems (47.5 ± 8.57). DLB patients showed the most IADL difficulties (41.8 ± 7.8) compared to PCA, typical AD, bvFTD, and PPA (adjusted β range 4.62 to 14.14, all *p* < 0.01), whereas PPA patients showed the least IADL difficulties (55.8 ± 9.83), with item‐specific differences.

**CONCLUSION:**

We found differences in everyday functioning between YOD types. Understanding IADL in YOD types will assist in care planning.

**Highlights:**

- Patients with DLB showed the most IADL difficulties compared to PCA, typical AD, bvFTD, and PPA
- Patients with PPA showed the least IADL difficulties compared to DLB, PCA, typical AD, and bvFTD
- We identified diagnostic group‐specific activity challenges. While ‘working’ was among the most commonly impaired activities across al groups, distinct functional challenges emerged per diagnosis: for example, DLB had high impairment in financial tasks, PCA patients in visual‐spatial tasks, and bvFTD with planning and organizational activities (e.g. making appointments).

## 1 BACKGROUND

Young‐onset dementia (YOD) refers to dementia with an onset occurring at <65 years of age. Alzheimer's disease (AD) and frontotemporal dementia (FTD) are considered to be the most prevalent underlying etiologies in YOD, with 41.1 and 2.3 per 100,000 population worldwide, respectively.^1^ These etiologies are often accompanied by an atypical clinical presentation, including more pronounced impairments in non‐memory domains such as language, executive functioning,^2^, ^3^ visuospatial abilities,^4^, ^5^, ^6^ and behavior.^7^ In particular, behavioral variant frontotemporal dementia (bvFTD) and YOD with Lewy bodies are often characterized by behavioral and neuropsychiatric symptoms.^8^ The clinical overlap of these syndromes with psychiatric disorders creates a diagnostic challenge, leading to a diagnostic delay of 4 to 5 years between symptom onset and diagnosis, compared to 2 to 3 years in late‐onset dementia (LOD).^9^ This delay has substantial social and financial consequences for patients and families.^10^, ^11^

An essential part of the diagnostic process is the extent to which cognitive impairments impact everyday functioning,^12^ which can be objectified using the construct of Instrumental Activities of Daily Living (IADL). This construct encompasses cognitively complex activities such as cooking, driving, working, and managing the household budget.^13^ Different measurement modalities exist, ranging from performance‐based tests, clinical interviews, and self‐reported to observer‐reported questionnaires. Regardless of the measurement modality, systematic reviews previously demonstrated substantial psychometric shortcomings in everyday functioning tools in the context of dementia.^14^, ^15^

The daily lives of individuals with YOD involve different everyday activities (e.g., continuing to work, maintaining active social and community roles) compared to LOD, which could lead to limited usefulness of existing tools assessing IADL. The Amsterdam‐IADL questionnaire (A‐IADL‐Q‐30)^13^, ^16^ was originally specifically developed for and validated in both LOD and YOD AD samples with input from individuals with dementia and their caregivers. Unlike many earlier IADL instruments that were mainly developed in a LOD population (e.g., DAD)^17^, the A‐IADL‐Q‐30 was designed to capture the full range of IADL in both YOD and LOD samples.^13^, ^18^ Moreover, it was recommended for use in dementia research, clinical practice, and core outcome sets.^19^, ^20^, ^21^ The use of the A‐IADL‐Q in this study offers a promising opportunity to examine disease‐specific IADL difficulties within a broad YOD spectrum, including multiple YOD types.

IADL performance varies across diagnostic groups. Ahmed et al.^22^ showed that posterior cortical atrophy (PCA) patients, assessed with the disability assessment for dementia (DAD) scale,^17^ had more pronounced IADL difficulties compared to typical AD. In addition, Mioshi et al.^23^ showed overall extensive IADL deficits assessed with the DAD, with behavioral variant frontotemporal dementia (bvFTD) having more impairments compared to subtypes of primary progressive aphasia (PPA) and AD. PPA showed least impairment, but these patients showed distinct difficulties in certain IADL items with a clear language component (e.g., use of telephone and managing finances), suggesting different patterns in IADL impairment. Morrow et al.^24^ confirmed these difficulties in language‐based activities, where impairments in “managing finances” and “meal preparation” were found in PPA.

These findings are difficult to compare, as IADL has been measured with different instruments, not always capturing all aspects of IADL and relying on measures with unclear psychometric qualities. Moreover, although these studies partially studied YOD patients, a direct comparison between YOD diagnostic subgroups is lacking, making it unclear to what extent and how these difficulties in everyday functioning differ between diagnostic groups across a broad YOD spectrum.

This study therefore aimed to (1) investigate IADL differences between YOD diagnostic groups and (2) investigate item‐level IADL differences between diagnostic groups using the A‐IADL‐Q.

## 2 METHODS

### 2.1 Study cohorts and inclusion criteria

In this cross‐sectional study, we included participants from two cohorts: 394 participants with a diagnosis of dementia due to typical AD (*n *= 261 [66%]), PCA (*n = *24 [6%]), dementia with Lewy bodies (DLB) (*n *= 25 [4%]), bvFTD (*n *= 49 [12%]), and PPA (*n *= 35 [9%]), from the memory‐clinic‐based Amsterdam Dementia Cohort (ADC)^25^ and 188 participants with a diagnosis of dementia due to AD (*n *= 176 [94%]), PCA (*n = *6 [3%]), and PPA (*n *= 6 [3%]) from the Longitudinal Early‐Onset Alzheimer's Disease Study (LEADS), a longitudinal observational study examining clinical, biological, and genetic characteristics related to disease progression in adults with young‐onset AD.^26^ The PPA group consisted of three clinical subtypes: semantic variant (svPPA, *n* = 19), non‐fluent/agrammatical variant (nfvPPA, *n* = 3), and logopenic variant (lvPPA, *n* = 14). Two participants did not meet criteria for any clinical subtype and were diagnosed with unclassified PPA (*n* = 2). Because of the relatively small number of cases per subtype, these were combined into a single group for the main analysis. PPA was subdivided into clinical subtype groups (svPPA and lvPPA) for exploratory purposes. The nvfPPA (*n* = 3) and unclassified PPA (*n* = 2) cases were excluded from the exploratory analyses due to insufficient sample size and, in the case of the unclassified PPA, lack of clinical subtype classification. Participants were recruited from memory clinics or research cohorts at LEADS sites in the United States. We selected individuals with (1) a diagnosis of dementia (AD, PCA, DLB, bvFTD, and PPA), (2) age of onset < 65 years, and (3) availability of IADL data.

### 2.2 Diversity, equity, and inclusion considerations

Participants were enrolled in both cohorts regardless of ethnic background to ensure diversity and equity in the design and execution of the study. Race and/or ethnicity was reported in the LEADS cohort and categorized as White, Hispanic, Black or African American, American Indian or Alaska Native, Native Hawaiian or Other Pacific Islander, Asian, or Other. In the ADC cohort, only ethnicity was reported and categorized as Caucasian, African, Hindustan, Moroccan, Turkish, or Other. Standardized procedures were used to ensure consistent data collection.

### 2.3 Diagnostic classification

For both the ADC and LEADS cohorts, clinical diagnoses were established through standardized criteria (see Apostolova 2021 et al.^26^ for LEADS procedures). For ADC, diagnoses were established in multidisciplinary consensus meetings based on a screening visit, including neurologic examination, neuropsychological assessment, and family history.

For both cohorts, diagnostic criteria followed the National Institute on Aging‐Alzheimer's Association (NIA‐AA)^27^ guidelines for AD, the crutch criteria for posterior cortical atrophy (PCA),^28^ and the Gorno–Tempini criteria for PPA.^29^ For ADC, DLB and bvFTD diagnoses were based on the international consensus diagnostic criteria for DLB^30^ and the international bvFTD criteria consortium for bvFTD.^31^

### 2.4 Ethical approval

The ADC study protocol was approved by the medical ethics review committee of the VU University Medical Center, and the LEADS protocol was approved by the Indiana University Central Institutional Review Board. All participants provided written informed consent. This study was conducted in accordance with the Declaration of Helsinki.^32^

**RESEARCH IN CONTEXT**

1. **Systematic review**: We examined differences in everyday functioning in YOD diagnostic groups and found IADL differences among groups, with DLB having the most difficulties and PPA the least.
2. **Interpretations**: Specific group differences were found, where DLB and AD had the most difficulty with “managing the household budget” and “paying bills,” PCA with “playing card and board games” and “driving a car,” bvFTD with “making appointments” and “making minor house repairs,” and PPA with “filling in forms” and “cooking.” These findings highlight a wide variety in IADL difficulties across YOD types that contribute to a deeper understanding of how impairments in everyday functioning manifest across neurodegenerative disorders.
3. **Future directions**: By integrating this knowledge into clinical practice, we can better align interventions with the specific needs of YOD patients and their families.

### 2.5 Measures

#### 2.5.1 Amsterdam IADL questionnaire

Everyday functioning was assessed using the 30‐item version of the A‐IADL‐Q (A‐IADL‐Q‐30).^16^ The A‐IADL‐Q‐30 is a study partner‐based questionnaire used to assess difficulties in performing cognitively complex activities (e.g., finances, working, and driving). The A‐IADL‐Q was previously found to have good content validity,^16^, ^33^ test–retest reliability (reliability coefficient of 0.97), construct validity,^13^ higher diagnostic accuracy compared to existing tests,^34^, ^35^ good cross‐cultural validity,^18^ and availability of normative data.^36^

Item scores were 0 (“no difficulty performing the activity”), 1 (“slightly more difficulty”), 2 (“more difficulty”), 3 (“much more difficulty”), and 4 (“no longer able to perform the activity”). Total scores (T‐score) were calculated using item response theory (IRT) modeling, taking difficulty levels and discrimination of different activities into account, with a score range from 20 to 80, with higher scores indicating better everyday functioning. In the A‐IADL‐Q‐30, missing responses (NA) indicate activities not applicable to the participant. IRT modeling estimates functioning based on the available items and is therefore robust to missing responses.^16^, ^33^ Compared to a traditional sum score, the IRT score was previously found to have higher sensitivity to mild deficits in everyday functioning.^37^

Subsequently, based on our previous mixed‐methods research on clinical meaningfulness, IADL scores were categorized into four levels, reflecting the level of impairment: no problems (T‐score ≥ 60), mild problems (T‐score 50 to 59), moderate problems (T‐score 40 to 49), and severe problems in IADL functioning (T‐score < 40).^38^

#### 2.5.2 Mini‐Mental State Examination

The Mini‐Mental State Examination (MMSE) was used to measure global cognitive abilities, with scores ranging from 0 to 30. The MMSE is used as a proxy for disease severity. Previous studies found good validity,^39^, ^40^ adequate internal consistency (Cronbach alphas > 0.71), high test–retest reliability (0.80 to 0.89), and good inter‐rater reliability (0.75).^41^, ^42^, ^43^ Higher scores indicate better cognitive functioning, where a score below 24 is considered abnormal.^41^

### 2.6 Statistical analyses

All statistical analyses were performed in R version 4.3.2.^44^ The packages dplyr, tibble, and readxl were used for structuring and combining datasets; MASS, car, and stats for statistical analysis; and ggplot2 and sjPlot for data visualization.

The ADC and LEADS datasets were merged to perform analyses on a combined dataset. Differences between diagnostic groups (i.e., typical AD, PCA, DLB, bvFTD, PPA) in MMSE, age, and education in years were tested with analyses of variance (ANOVA), and differences in sex were tested with chi‐squared tests. For ADC, education levels classified according to the Dutch Verhage System were converted to education in years as an intermediate step.^45^, ^46^ For both cohorts (ADC and LEADS) education in years was used.

To test differences in IADL between diagnostic groups, multiple linear regression analyses were performed, with IADL total scores as a continuous outcome measure and the diagnostic group as the independent variable (changing the reference group to achieve pairwise comparisons). Three models were run to examine the differences in IADL total scores across diagnostic groups: (1) univariable model; (2) model adjusted for age, sex, MMSE, and education; (3) model adjusted for sex, cohort, age, MMSE, and education. The assumptions for multiple linear regressions were met: linearity, independence of errors, homoscedasticity, normality of errors, no multicollinearity, and no significant outliers.

To explore diagnostic group‐specific difficulties at the item level, item endorsement counts were computed by calculating the frequency of endorsements for each item within each diagnostic group. Missing responses were defined as NA. Next, percentages of responses in category 4 (= no longer able to perform the task) and category 0 (= no problems) were calculated to detect the most and least affected activities for each diagnostic group. Multiple ordinal logistic regression analyses (pairwise comparisons) were used to analyze the association between the diagnostic group as the predictor (nominal categorical variable) and scores on each item as the outcome (as an ordinal variable). Odds ratios (ORs) were computed to compare the likelihood of item endorsement between the diagnostic groups and were reported as effect measures. Second, ORs were ranked in ascending order within the diagnostic group, to identify the top five items with the highest ORs. For visualization, items were ordered in ascending order based on the percentage of participants in response category 4 (= no longer able to perform the task).

## 3 RESULTS

The demographic characteristics of the study sample, stratified by diagnostic group, are displayed in Table 1.

**TABLE 1:** Demographic characteristics per diagnostic group.

	Total group	Typical AD	PCA	DLB	bvFTD	PPA	*p* value
***n*** **(%)**	582	437 (75)	30 (4)	25 (4)	49 (8)	41 (7)	
**Female sex** **(%)**	345 (59)	229 (52)	22 (73)	6 (24)	24 (49)	18 (44)	0.006^b^
**Age**	58.38 ± 4.3	58.14 ± 4.1	58.60 ± 4.2	62.08 ± 1.9^***^	58.20 ± 5.6^**^	58.68 ± 4.5^*^	<0.001^a^
**Years after onset**	3.22 ± 2.27	3.34 ± 2.41	3.30 ± 1.58	2.75 ± 1.33	2.90 ± 2.29	2.55 ± 1.93	0.174
**Education in years**	12.74 ± 3.3	13.06 ± 3.4	12.13 ± 2.66	12.08 ± 2.8	10.78 ± 2.7^***^	12.56 ± 3.1	<0.001^a^
**MMSE**	20.84 ± 5.6	20.32 ± 5.5	20.37 ± 4.9	22.91 ± 4.2	23.40 ± 5.0^**^	22.74 ± 6.5	<0.001^a^
**Cohorts**, ***n*** **(%)**							
ADC	394 (68)	261 (66)	24 (6)	25 (6)	49 (12)	35 (9)	
**Amyloid confirmation (CSF A+/T+), *n* (%)**	208 (52.8)	182 (69.7)	18 (75)	4 (16)	0 (0)	4 (11.4)	
LEADS	188 (32)	176 (94)	6 (3)	0 (0)	0 (0)	6 (3)	

*Note*: Shown here are mean (M) and standard deviations (SD) for sex, age, years after onset, education, and MMSE, stratified per diagnostic group. Post hoc differences are indicated by asterisks (*), for age.

Abbreviations: ADC, Amsterdam Dementia Cohort; bvFTD, behavioral variant frontotemporal dementia; CSF, cerebrospinal fluid; DLB, dementia with Lewy bodies; MMSE, Mini‐Mental State Examination; PCA, posterior cortical atrophy; PPA, primary progressive aphasia; Typical AD, typical Alzheimer's disease.

^a^ Tested with ANOVA.

^b^ Tested with chi‐squared test.

***DLB vs. typical AD (*p* < 0.01), for age.

**bvFTD vs.DLB (*p* < 0.01), for age.

*PPA vs. DLB (*p* < 0.05), for age.

***bvFTD vs. typical AD (*p* < 0.01), for education.

**bvFTD vs. typical AD (*p* < 0.01), for MMSE.

In the total cohort, patients with dementia due to typical AD were most represented (*n *= 437 [75%]), followed by bvFTD (*n *= 49 [8%]), PPA (*n *= 41 [7%]), PCA (*n = *30 [5%]), and DLB (*n *= 25 [4%]). Dementia subgroups differed in MMSE scores (*F*(4, 559) = 5.5, *p* < 0.001), with bvFTD having the highest MMSE (23.4 ± 5.0) and typical AD the lowest MMSE scores 20.3 ± 5.5. The diagnostic groups did not differ on years after onset (*p* > 0.05). Post hoc analyses showed specific group differences for age, education, and years, as depicted in Table 1.

Appendix A, Table A1, details a breakdown of demographic characteristics by cohort.

The LEADS cohort (15.55 ± 2.4) had a higher mean years of education (11.40 ± 2.7, *p* < 0.001) and a higher mean years after onset (3.82 ± 2.27, *p* < 0.001) than the ADC cohort (Appendix A, Table A1). There were no significant differences between the ADC cohort and LEADS cohort regarding age, sex, and MMSE. Participants in both cohorts were predminantly White (LEADS [85%] and ADC [89%]). In our data, percentage of missing responses on the A‐IADL‐Q‐30 (NA, for “not applicable”) was 34%.

### 3.1 IADL difficulties

**FIGURE 1:**
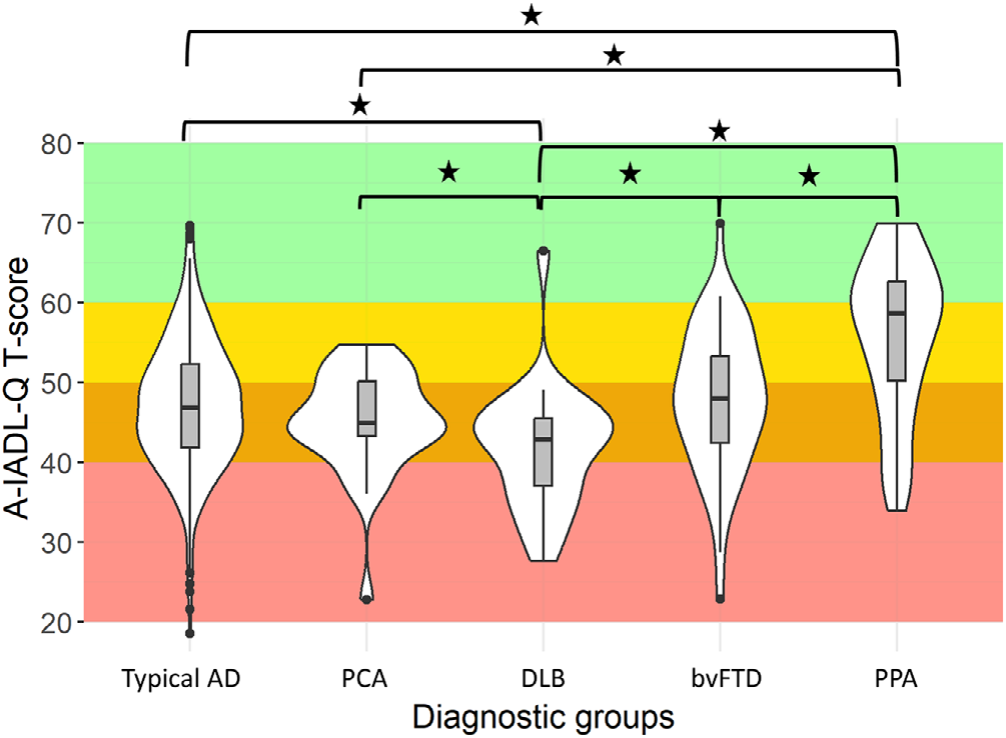
Differences in Instrumental Activities of Daily Living distributions for diagnostic groups. *Note*: Shown here are distributions of IADL T‐scores for the separate diagnostic groups. Color‐coded classifications: red = severe problems, orange = moderate problems, yellow = mild problems, green = no problems, according to clinical meaningful categorization of Dubbelman et al. (2020). Significant differences are indicated by an asterisk (*). These results are based on the univariable model. bvFTD, behavioral variant fron to temporal dementia; DLB, dementia with lewy bodies; PCA, posterior cortical atrophy; PPA, primary progressive aphasia; Typical AD, typical alzheimer's disease; IADL, instrumental activities of daily living.

The prevalence of problems in everyday functioning (A‐IADL‐Q T‐score ≤ 60) in the total sample was 92.4%, compatible with the diagnosis of dementia. The overall sample showed moderate problems in everyday functioning (A‐IADL‐Q 47.5 ± 8.6). Differences in A‐IADL‐Q T‐scores between groups indicated that DLB had the most IADL difficulties, while PPA group experienced the fewest IADL difficulties (Figure 1).

Multiple linear regression analyses, adjusted for age, sex, education, cohort, and MMSE (fully adjusted model), confirmed that DLB had the lowest IADL score (A‐IADL‐Q 41.8 ± 7.8), reflecting most IADL difficulties when compared to other diagnostic groups. Specifically, DLB exhibited greater impairments compared to PCA (A‐IADL‐Q 45.2 ± 6.5, *p* < 0.02), typical AD (A‐IADL‐Q 47.2 ± 8.1, *p* < 0.001), bvFTD (A‐IADL‐Q 47.9 ± 9.0, *p* < 0.001), and PPA (A‐IADL‐Q 55.8 ± 9.83, *p* < 0.001). On the other hand, PPA had the least IADL difficulties, and post hoc comparisons showed less impairment compared to typical AD (*p* < 0.001), PCA (*p* < 0.001), DLB (*p* < 0.001), and bvFTD (*p* < 0.001). The results from the unadjusted and adjusted models using typical AD as the reference group are provided in Table 2. All pairwise comparisons between diagnostic groups are presented in Appendix A, Table A2.

**TABLE 2 alz70711-tbl-0002:** Multiple regression analysis results of Instrumental Activities of Daily Living functioning for diagnostic groups.

Diagnostic group	Model 1 *n* = 582	Model 2 *n* = 564	Model 3 *n* = 564
**PCA**	−2.03 [−5.08, 1.01]	−2.22 [−4.84, 0.4]	−2.10 [−4.73, 0.52]
**DLB**	−5.35 [−8.67, 2.04]	−6.99 [−10.02, 3.96]	−6.72 [−9.8, 3.65]
**bvFTD**	0.74 [−1.69, 3.17]	−0.72 [−2.95, 1.50]	−0.55 [−2.81, 1.70]
**PPA**	8.65 [6.02 to 11.28]	7.24 [4.92 to 9.56]	7.41 [5.07 to 9.76]

*Note*: Results are presented as beta values with 95% confidence interval. For the univariable model (unadjusted model 1), adjusted for age, sex, MMSE, and education (adjusted model 2) and adjusted for age, sex, MMSE, education, and cohort (fully adjusted model 3). Typical Alzheimer's disease (AD) was used as the reference group in all models.

Abbreviations: bvFTD, behavioral variant frontotemporal dementia; DLB, dementia with Lewy bodies; IADL, Instrumental Activities of Daily Living; PCA, posterior cortical atrophy; PPA, primary progressive aphasia; SE, standard error; Typical AD, typical Alzheimer's disease.

Exploratory analyses of the PPA clinical subtypes, adjusted for age, sex, education, cohort, and MSME (fully adjusted model), suggested that both svPPA and lvPPA were associated with fewer IADL difficulties compared to typical AD (*p* < 0.001), PCA (*p* < 0.001), DLB (*p* < 0.001), and bvFTD (*p* < 0.001). The direction of effect was similar for both PPA subtypes: svPPA (*β* range: 8.29 to 14.49) and lvPPA (*β* range: 5.84 to 12.03). However, the difference between svPPA and lvPPA did not reach statistical significance (*p* > 0.001) (full results in Table A3, Appendix A).

Item‐level analysis showed that when inspecting the activities for which “severe problems” (i.e., “no longer able to perform activity”) were reported across diagnostic groups, “working” ranked consistently among the top five most difficult activities, ranging from 19% of PPA participants to 61% of DLB participants no longer able to perform the activity (*χ* ^2^ = 32.51, *p* < 0.01).

When inspecting group‐specific activities for which “severe problems” were reported (i.e., activities with the highest percentages of severe problems), some group‐specific patterns emerged. In typical AD and DLB, tasks related to “managing the household budget” (40% in AD, 71% in DLB; *χ* ^2^ = 40.09, *p* < 0.01), and “paying bills” (36% in AD, 61% in DLB; *χ* ^2^ = 48.04, *p* < 0.001) were most frequently severely impaired. For PCA, most prevalent severe impairments were observed in “managing the household budget” (55%, *χ* ^2^ = 40.09, *p* < 0.01), “driving a car” (46%, *χ* ^2^ = 45.22, *p* < 0.01), and “playing card and board games” (38%, *χ* ^2^ = 34.26, *p* < 0.01). In bvFTD, “making appointments” (43%, *χ* ^2^ = 80.98, *p* < 0.001) and “making minor house repairs” (42%, *χ* ^2^ = 35.46, *p* < 0.01) were most often reported to be severely impaired. For PPA, “filling in forms” (16%, *χ* ^2^ = 32.14, *p* < 0.01) and “cooking” (14%, *χ* ^2^ = 45.84, *p* < 0.001) had the highest rates of severe difficulty.

When focusing on the least impaired activities (based on the proportion reporting no difficulty) across diagnostic groups, “preparing sandwich meals” ranked among the top five least difficult activities (44% to 87%, *χ* ^2^ = 42.04, *p* < 0.001), and “operating the coffee maker” was similarly less challenging for PCA, DLB, bvFTD, and PPA (64% to 80%, *χ* ^2^ = 48.65, *p* < 0.001). All item‐level prevalence data per diagnostic group are presented in[Table alz70711-tbl-0002] Figure 2.

FIGURE 2Prevalence of Amsterdam Instrumental Activities of Daily Living questionnaire item responses for different diagnostic groups. *Note*: A‐IADL‐Q item responses (%) are shown in ascending order for each item of the A‐IADL‐Q, stratified by diagnostic group. A‐IADL‐Q, amsterdam IADL questionnaire; IADL, instrumental activities of daily living; Typical AD, typical alzheimer's disease; PCA, posterior cortical atrophy; DLB, dementia with lewy bodies; bvFTD, behavioral variant frontotemporal dementia; PPA, primary progressive aphasia.

**Figure:**
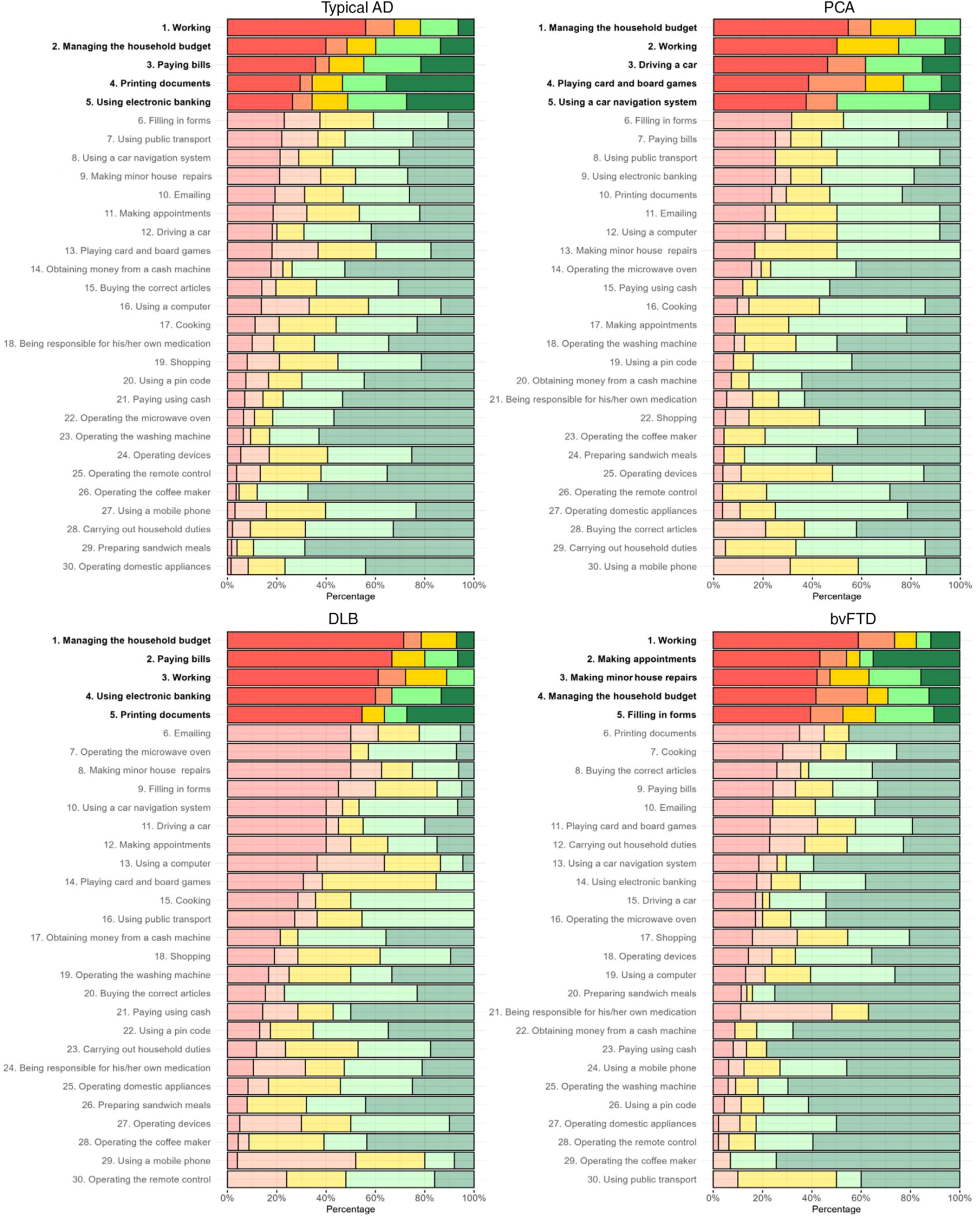

**Figure:**
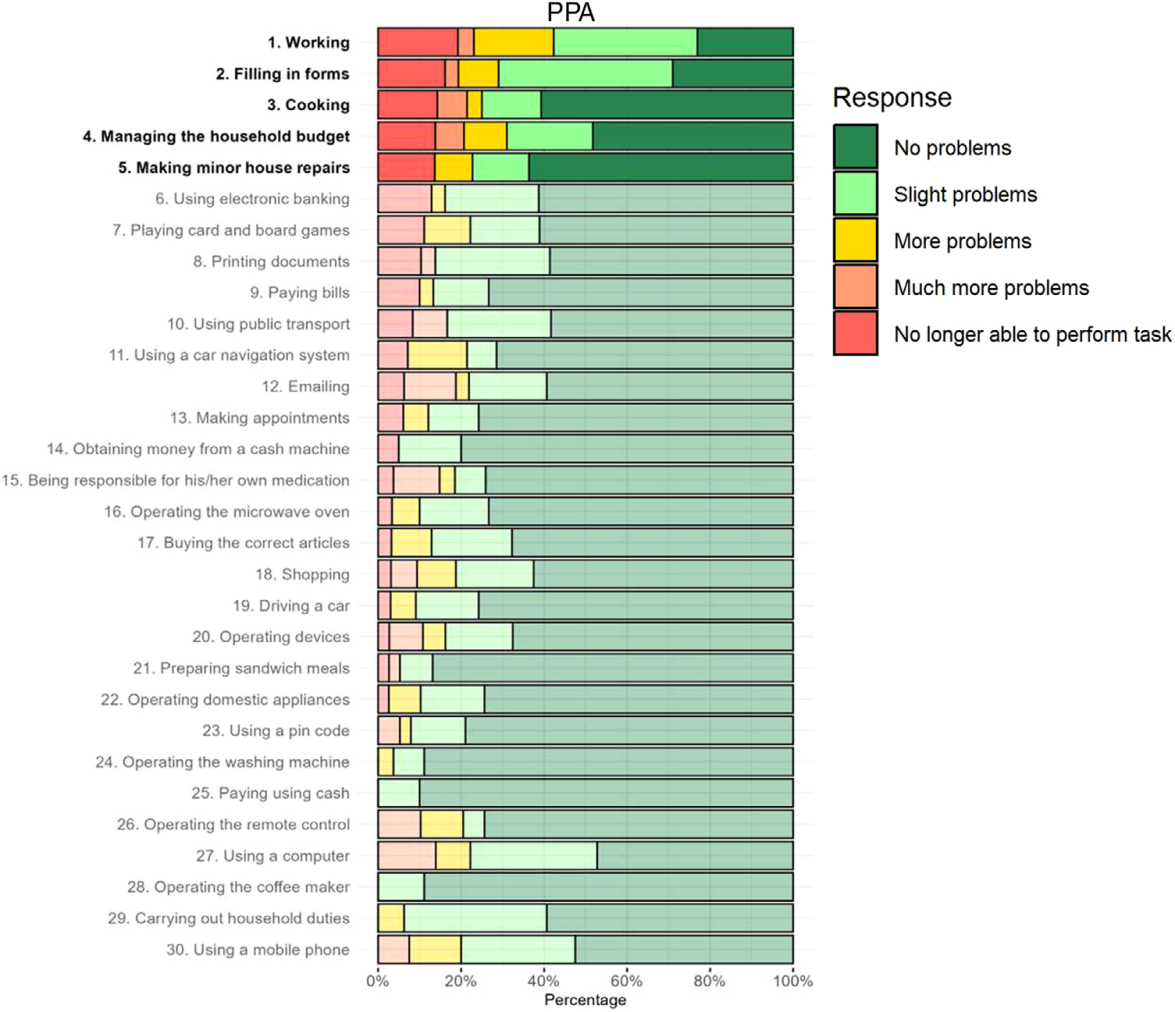

These item‐level differences were supported by ordinal regression analysis. Compared to the least impaired group (PPA, used as the reference group), higher odds for difficulties in “making appointments” were found in typical AD, DLB, and bvFTD (OR range 9.8 to 20.2; Table A4, Appendix A).

Additional group‐specific comparisons showed higher odds for increasing difficulty in “paying with cash” in typical AD (OR = 7.9, 95% confidence interval [CI]: 2.3, 50.4), in “driving a car” for PCA (OR = 17.7, 95% CI: 5.0, 67.1), in “operating the microwave oven” for DLB (OR = 19.7, 95% CI: 5.6, 78.0), and in “carrying out household duties” for bvFTD (OR = 11.7, 95% CI: 4.5, 31.5). Full results are presented in Table A4, Appendix A.

## 4 DISCUSSION

In this study, we found considerable variability in IADL problems between different YOD types. DLB showed the most problems in everyday functioning and PPA the least, with AD, PCA, and bvFTD in between. On the item level, DLB and AD had the most difficulty with “managing the household budget” and “paying bills,” PCA with “playing card and board games” and “driving a car,” bvFTD with “making appointments” and “making minor repairs to the house,” and PPA with “filling in forms” and “cooking.”

The overall findings of differences in everyday functioning between YOD diagnostic groups align with previous literature in both YOD and LOD.^23^, ^47^ Similar to our study, Mioshi et al.^23^ described bvFTD performing worse on IADL compared to PPA and typical AD. Mioshi et al. (2007) included only svPPA and nfvPPA, whereas our study also included lvPPA, which is an important addition given its faster disease progression.^29^ Since DLB was not included in this study, we build upon these findings by including more diagnostic groups, which contributes to a more complete representation of the YOD spectrum and its functional implications. Moreover, we adjusted for disease severity, which highlights the presence of differences irrespective of disease stage. Our findings of DLB having more IADL difficulties are in line with previous work in LOD, which showed that DLB had more difficulties compared to AD.^47^, ^48^ In LOD, a potential explanation for the higher level of IADL impairment in DLB was the presence of hallucinations and poor motor functioning.^47^, ^48^, ^49^ This might also be the case in YOD, although the specific affected difficulties observed in DLB (“managing the household budget” and “paying bills”) could also indicate impairments in non‐memory domains, such as attention, visuospatial abilities, and executive functions.

Moreover, by the use of the widely validated A‐IADL‐Q with good psychometric properties, we were able to better capture difficulties in everyday functioning. This allows for a more precise understanding of the impact of YOD on everyday functioning. Hendriks et al.^6^ retrospectively extracted from general practitioner notes that do not always include IADL issues documented in a consistent and standardized manner and were not assessed using a reliable and widely validated psychometric scale. Mioshi et al.^23^ used the DAD, a scale developed for LOD and primarily focused on traditional IADL and therefore possibly less responsive to early cognitive changes and modern tasks, such as technology use.^34^ Using the A‐IADL‐Q, we assessed everyday functioning more accurately in YOD and made more reliable comparisons between diagnostic groups.

Our activity‐level evaluation is consistent with previous studies examining differences in everyday functioning within atypical dementia, as demonstrated by Mioshi et al.^23^ and Morrow et al.,^24^ where impairments in managing finances and meal preparation were found in PPA. These findings suggest that underlying cognitive mechanisms are responsible for the everyday functioning impairment. For example, at the item level, we found that individuals with bvFTD have the most difficulty in activities such as “making appointments” and “making minor house repairs.” One might speculate that these are in line with executive function disturbances, which were commonly found in the atypical presentation of YOD,^4^, ^50^ affecting planning, problem‐solving, and decision‐making abilities.^5^, ^6^, ^51^, ^52^ Similarly, PCA showed the most difficulty with driving a car and playing card and board games, which could be understood as activities with a visual component, which is in line with the cognitive clinical presentation of PCA.^28^ Van der Landen et al.^53^ confirmed this association between IADL and visuospatial abilities. Our study highlights the unique challenges faced by different YOD diagnostic groups and contributes to the literature by examining item‐level differences in everyday functioning, particularly in atypical presentations of dementia.

The limitations of the current study that need to be taken into consideration include the relatively small sample sizes for the specific atypical dementia subgroups compared to the typical AD sample. Even though this reflects the YOD diagnostic distribution, such unequal sample sizes could have affected the statistical power and reliability of the results.^54^ However, this does not compromise the validity of the regression estimates. Multiple linear regression can accommodate unequal sample sizes without introducing bias into the coefficient estimates.^55^ Second, we merged data from two cohorts with different recruitment methods: the memory‐clinic‐based ADC and the LEADS study, which included participants from both memory clinics and research cohorts, resulting in slight differences in composition. More specifically, the LEADS cohort only included a limited number of atypical presentations. This led to limitations in the use of more advanced statistical methods to adjust for the nested data. Third, we used the MMSE as a proxy for disease severity in the absence of other disease staging measures. However, the MMSE is a cognitive measure and is not a reflection of the actual disease stage – particularly not in specific YOD diagnostic groups such as PPA, where the MMSE could overestimate the actual disease severity.^56^ Nonetheless, our results revealed that the diagnostic groups did not differ with respect to years after onset, which suggests the groups are at a comparable stage of disease progression at their baseline visit. Fourth, we combined the PPA subtypes (semantic variant [svPPA], non‐fluent/agrammatical variant [nfvPPA], and logopenic variant [lvPPA]), and unclassified PPA into a single PPA group because of the relatively small sample sizes for each subtypes, resulting in more statistical power for the analyses. However, the different etiologies of the subtypes might have different profiles in everyday functioning. Therefore, we conducted exploratory analyses by subdividing the PPA group into svPPA and lvPPA diagnostic subgroups, excluding nfvPPA and unclassified PPA due to small sample sizes and, in the latter case, the absence of a classification. As a result, these findings do not reflect the full PPA spectrum and should be interpreted with caution. The results showed similar results compared to the main analyses, in that both subtypes demonstrated fewer IADL difficulties compared to the other diagnostic groups. svPPA showed a slightly stronger trend toward preserved everyday functioning than lvPPA, although this difference was not statistically significant. These findings suggest that differences in everyday functioning may exist between svPPA and lvPPA, but larger samples (including all three PPA subtypes) are needed to explore this further. Fifth, the sample was predominantly White, which may limit the generalizability of the results to the broader population, including minority populations. Future studies should focus on recruitment strategies that target these populations to improve generalizability of dementia research.

The strengths of this study include the comparison of diagnostic groups on a broad YOD spectrum (onset < 65 years of age) and the use of a widely validated IADL questionnaire, which may serve as a diagnostic tool to systematically and possibly earlier than otherwise detect impairments in everyday functioning. Contrary to the A‐IADL‐Q, studies have shown that other IADL questionnaires lack good psychometric quality.^14^

The implications of this study are as follows. With the collected IADL in YOD data, using a consistent reliable measurement instrument, we were able to make meaningful comparisons across groups. These findings can inform more tailored care strategies for the specific YOD types, providing information on everyday functioning specific to each diagnostic group and outlining potential challenges that patients and caregivers may face in the future. Furthermore, future research should focus on examining earlier stages of the disease,^6^ perform longitudinal assessments of functional decline across atypical dementias, and explore the relationship between cognition and daily functioning to refine intervention and support strategies.

To conclude, we observed substantial variability in IADL impairments across different YOD types, with distinct differences in activity‐level challenges among diagnostic groups. By incorporating the A‐IADL‐Q, we provide new insights into the specific ways YOD impacts everyday functioning, offering valuable information for patients, caregivers, and clinicians. These findings can help guide more tailored care strategies and interventions, better aligning with the unique needs of YOD patients and their families.

## CONFLICT OF INTEREST STATEMENT

None of the authors involved in this project have disclosed any conflicts of interest that could affect the findings. Author disclosures are available in the supporting information.

## CONSENT STATEMENT

All authors have reviewed and given their consent to be listed as contributors to this manuscript.

## Supporting information



Supporting Information

Supporting Information

Supporting Information
