# Supplementary material for: Everyday functioning in young onset dementia: differences between diagnostic groups
Source: Alzheimers Dement. 2025 Sep 24;21(9):e70711. doi: 10.1002/alz.70711 (PMC12458908; doi:10.1002/alz.70711)
Supplement: Supplementary file 1 — Supporting Information [file ALZ-21-e70711-s001.docx]

**APPENDIX A**

**TABLE A.1** Demographic characteristics per cohort

|  | **Cohort**  **LEADS (n = 188)** | **ADC (n =394)** | ***P* value** |
| --- | --- | --- | --- |
| **Demographics** | 188 | 394 |  |
| Female gender, n (%) | 105 (56) | 194 (49) | 0.160^b^ |
| Age | 58.29 ± 4.04 | 58.42 ± 4.0 | 0.720^a^ |
| Years post-onset | 3.82 ± 2.27 | 2.97 ± 2.26 | <0.001^a^ |
| Education in years | 15.55 ± 2.4 | 11.40 ± 2.7 | <0.001^a^ |
| MMSE | 20.33 ± 5.7 | 21.08 ± 5.5 | 0.134^a^ |
| **Race/ethnicity (%)** |  |  |  |
| Non-Hispanic White/Caucasian | 81 | 89 |  |
| Hispanic White | 4 | _ |  |
| Black or African American | 9 | _ |  |
| African | _ | 3 |  |
| Asian | 4 | _ |  |
| Marroccan | _ | 1 |  |
| Turkish | _ | 2 |  |
| Other | 2 | 6 |  |

**Note:** Shown here are mean (M) and standard deviations (SD) for gender, age, race, years post-onset, education and MMSE, and percentages (%) for race/ethnicity, stratified by cohort. Abbreviations: ADC Amsterdam Dementia Cohort LEADS Longitudinal Early Onset Alzheimer’s Disease Study. ^a^ tested with t-test, ^b^ tested with Chi-squared test

| **Diagnostic group** | Reference group  **Typical AD** | **PCA** | **DLB** | **bvFTD** | **PPA** |
| --- | --- | --- | --- | --- | --- |
| **Model 1 (N = 582)** |  |  |  |  |  |
| Typical AD | ­_ | 2.03 [-1.01-5.08] | 5.35 [2.04-8.67] | -0.74 [-3.17-1.69] | -8.65 [-11.28- -6.02] |
| PCA | -2.03 [-5.08-1.01] | _ | 3.32 [-1.05-7.68] | -2.78 [-6.51-0.96] | -10.68 [-14.56- 6.81] |
| DLB | -5.35 [-8.67- -2.04] | -3.32 [-7.68-1.05] | _ | -6.09 [-10.06- -2.13] | -14.00 [-18.09- -9.91] |
| bvFTD | 0.74 [-1.69-3.17] | 2.78 [-0.96-6.51] | 6.09 [2.13-10.06] | _ | -7.91 [-11.32- 4.5] |
| PPA | 8.65 [6.02-11.28] | 10.68 [6.81-14.56] | 14.00 [9.91-18.09] | 7.91 [4.50-11.32] | _ |
| **Model 2 (N = 564)** |  |  |  |  |  |
| Typical AD | _ | 2.22 [-0.4-4.84] | 6.99 [3.96-10.02] | 0.72 [-1.50- 2.95] | -7.24 [-9.56- -4.92] |
| PCA | -2.22 [-4.84-0.4] | _ | 4.77 [0.88-8.67] | -1.50 [-4.78- 1.79] | -9.46 [-12.83- -6.09] |
| DLB | -6.99 [-10.02- -3.96] | -4.77 [-8.67- -0.88] | _ | -6.27 [-9.85- -2.68] | -14.23 [-17.9- 10.57] |
| bvFTD | -0.72 [-2.95-1.50] | 1.50 [-1.79-4.78] | 6.27 [2.68-9.85] | _ | -7.97 [-11.0- -4.93] |
| PPA | 7.24 [4.92-9.56] | 9.46 [6.09-12.83] | 14.23 [10.57-17.9] | 7.97 [4.93-11.0) | _ |
| **Model 3 (N = 564)** |  |  |  |  |  |
| Typical AD | _ | 2.10 [-0.52-4.73] | 6.72 [3.65-9.8] | 0.55 [-1.70-2.81] | -7.41 [-9.76- -5.07] |
| PCA | -2.10 [-4.73-0.52] | _ | 4.62 [0.71-8.52] | -1.55 [-4.84-1.74] | -9.52 [-12.89- -6.14] |
| DLB | -6.72 [-9.8- -3.65] | -4.62 [-8.52- -0.71] | _ | -6.17 [-9.76- -2.58] | -14.14 [-17.8- -10.47] |
| bvFTD | -0.55 [-2.81-1.70] | 1.55 [-1.74-4.84] | 6.17 [2.58-9.76] | _ | -7.97 [-11.0- -4.93] |
| PPA | 7.41 [5.07-9.76] | 9.52 [6.14-12.89] | 14.14 [10.47-17.8] | 7.97 [4.93-11.0] | _ |

**TABLE A.2.** Multiple regression analysis results of IADL functioning for diagnostic groups

**Note:** Results are presented as beta values with 95% confidence interval. for the univariable model (unadjusted model 1), adjusted for age, sex, MMSE, and education (adjusted model 2), and adjusted for age, sex, MMSE, education, and cohort (fully adjusted model 3). Abbreviations: IADL instrumental activities of daily living, SE Standard Error, Typical AD Typical Alzheimer’s Disease, PCA Posterior Cortical Atrophy, DLB Dementia with Lewy Bodies, bvFTD behavioral variant frontotemporal dementia, PPA Primary Progressive Aphasia.

**TABLE A.3.** Exploratory multiple regression analysis results of IADL functioning for diagnostic groups, including PPA subtypes

| **Diagnostic group** | Reference group  **Typical AD** | **PCA** | **DLB** | **bvFTD** | **lvPPA** | **svPPA** |
| --- | --- | --- | --- | --- | --- | --- |
| **Model 1 (N = 577)** |  |  |  |  |  |  |
| Typical AD | ­_ | 2.03 [-0.99-5.05] | 5.35 [2.06-8.64] | -0.74 [-3.15-1.67] | -5.87 [-9.95- -0.52] | -9.55 [-13.21- -5.89] |
| PCA | -2.03 [-5.05-0.99] | _ | 3.32 [-1.02-7.65] | -2.78 [-6.49-0.93] | -7.91 [-12.86- -2.95] | -11.58 [-16.20- -6.96] |
| DLB | -5.35 [-8.67- -2.06] | -3.32 [-7.65-1.01] | _ | -6.09 [-10.03- -2.16] | -11.22 [-16.35- -6.10] | -14.99 [-19.70 - -10.10] |
| bvFTD | 0.74 [-1.67-3.15] | 2.78 [-0.93-6.49] | 6.09 [2.16-10.03] | _ | -5.13 [-9.74- -0.52] | -8.80 [-13.05- -4.56] |
| lvPPA | 5.87 [1.80-9.95] | 7.90 [2.95-12.86] | 11.22 [6.10-16.35] | 5.13 [0.52-9.74] | _ | -3.67 [-9.04- -1.69] |
| svPPA | 9.55 [5.89-12.21] | 11.58 [6.96-16.20] | 14.90 [10.10-19.70] | 8.80 [4.56-13.05] | 3.67 [-1.69-9.04] | _ |
| **Model 2 (N = 559)** |  |  |  |  |  |  |
| Typical AD | _ | 2.18 [-0.43-4.80] | 6.93 [3.90-9.95] | 0.63 [-1.60- 2.85] | -5.29 [-8.80- -1.78] | -7.62 [-10.98- -4.26] |
| PCA | -2.18 [-4.80-0.43] | _ | 4.74 [0.85-8.63] | -1.56 [-4.84- 1.72] | -7.47 [-11.76- -3.18] | -9.80 [-13.95- -5.65] |
| DLB | -6.93 [-9.95- -3.90] | -4.74 [-8.63- -0.85] | _ | -6.30 [-9.88- -2.72] | -12.21 [-16.72- -7.70] | -14.54 [-18.92- -10.16] |
| bvFTD | -0.63 [-2.85-1.60] | 1.56 [-1.72-4.84] | 6.30 [2.72-9.88] | _ | -5.91 [-9.96- -1.86] | -8.24 [-12.08- -4.40] |
| lvPPA | 5.29 [1.78-8.80] | 7.47 [3.19-11.76] | 12.21 [7.70-16.72] | 5.91 [1.86-9.96] | _ | -2.33 [-7.09-2.44] |
| svPPA | 7.62 [4.26-10.98] | 9.80 [5.65-13.95] | 14.54 [10.16-18.92] | 8.24 [4.40-12.08] | 2.33 [-2.44- 7.09] | _ |

**TABLE A.3 (continued).** Exploratory multiple regression analysis results of IADL functioning for diagnostic groups, including PPA subtypes

| **Model 3 (N = 559)** |  |  |  |  |  |  |
| --- | --- | --- | --- | --- | --- | --- |
| Typical AD | _ | 2.05 [-0.57-4.68] | 6.72 [3.56-9.69] | 0.43 [-1.82-2.68] | -5.41 [-8.92- -1.89] | -7.86 [-11.24- -4.48] |
| PCA | -2.05 [-4.68-0.57] | _ | 4.57 [0.67-8.47] | -1.62 [-4.91-1.66] | -7.46 [-11.75- -3.18] | -9.92 [-14.07- -5.76] |
| DLB | -6.62 [-9.69- -3.56] | -4.57 [-8.47- -0.67] | _ | -6.19 [-9.79- -2.61] | -12.03 [-16.55- -7.51] | -14.49 [-18.87- -10.10] |
| bvFTD | -0.43 [-2.68-1.82] | 1.62 [-1.66-4.91] | 6.19 [2.60-9.78] | _ | -5.84 [-9.89- -1.79] | -8.29 [-12.13- -4.45] |
| lvPPA | 5.41 [1.89-8.92] | 7.46 [3.18-11.75] | 12.03 [7.51-16.55] | 5.84 [1.79-9.89] | _ | -2.45 [-7.22-2.32] |
| svPPA | 7.86 [4.48-11.24] | 9.92 [5.76-14.07] | 14.49 [10.10-18.87] | 8.29 [4.45-12.13] | 2.45 [-2.31- 7.22] | _ |

**Note:** Results are presented as beta values with 95% confidence interval. for the univariable model (unadjusted model 1), adjusted for age, sex, MMSE, and education (adjusted model 2), and adjusted for age, sex, MMSE, education, and cohort (fully adjusted model 3). Abbreviations: IADL instrumental activities of daily living, SE Standard Error, Typical AD Typical Alzheimer’s Disease, PCA Posterior Cortical Atrophy, DLB Dementia with Lewy Bodies, bvFTD behavioral variant frontotemporal dementia, lvPPA logopenic variant Primary Progressive Aphasia, svPPA semantic variant Primary Progressive Aphasia.

**TABLE A.4** Top five most difficult A-IADL-Q items per diagnostic group

| **Diagnosis** | **Item** | **OR [95% CI]** |
| --- | --- | --- |
| **Typical AD** | Making appointments | 9.8 [4.5, 23.9] |
|  | Paying bills | 9.3 [4.1, 23.2] |
|  | Paying with cash | 7.9 [2.3, 50.4] |
|  | Playing card and board games | 6.5 [2.5, 18.5] |
|  | Operating devices | 5.4 [2.7, 11.4] |
| **PCA** | Driving a car | 17.7 [5.0, 67.1] |
|  | Playing card and board games | 16.9 [4.3, 70.6] |
|  | Managing the household budget | 10.0 [2.8, 39.5] |
|  | Using a car navigation system | 9.9 [2.3, 46.4] |
|  | Operating the coffee maker | 9.9 [3.0, 39.4] |
| **DLB** | Paying bills | 31.9 [8.8, 132.4] |
|  | Making appointments | 20.2 [6.6, 66.4] |
|  | Operating the microwave oven | 19.7 [5.6, 78.0] |
|  | Managing the household budget | 18.8 [5.2, 80.8] |
|  | Using a computer | 18.77 [7.0, 51.5] |
| **bvFTD** | Making appointments | 16.2 [5.8, 48.3] |
|  | Carrying out household duties | 11.7 [4.5, 31.5] |
|  | Being responsible for medication | 8.7 [2.9, 27.9] |
|  | Making minor repairs to the house | 8.6 [2.6, 29.8] |
|  | Shopping | 8.2 [3.3, 20.7] |

**Note:** Odds Ratio’s (OR) for top items with most difficulty are shown per diagnostic group compared to PPA as reference group. *Abbreviations: IADL* instrumental activities of daily living, *Typical AD* Typical Alzheimer’s Disease*, PCA* Posterior Cortical Atrophy*, DLB* Dementia with Lewy Bodies*, bvFTD* behavioral variant frontotemporal dementia, *95% CI* 95% confidence interval
