# Supplementary material for: Everyday functioning in young onset dementia: differences between diagnostic groups
Source: Alzheimers Dement. 2025 Sep 24;21(9):e70711. doi: 10.1002/alz.70711 (PMC12458908; doi:10.1002/alz.70711)
Supplement: Supplementary file 3 — Supporting Information [file ALZ-21-e70711-s002.docx]

**Highlights**

- Patients with DLB showed the most IADL difficulties compared to PCA, typical AD, bvFTD, and PPA
- Patients with PPA showed the least IADL difficulties compared to DLB, PCA, typical AD, and bvFTD
- We identified diagnostic group-specific activity challenges. While ‘working’ was among the most commonly impaired activities across al groups, distinct functional challenges emerged per diagnosis: for example, DLB had high impairment in financial tasks, PCA patients in visual-spatial tasks, and bvFTD with planning and organizational activities (e.g. making appointments).
